# Hepatic Tuberculosis Manifested as Hepatic Abscess: A Report of a Unique Case

**DOI:** 10.7759/cureus.53094

**Published:** 2024-01-28

**Authors:** Nilesh Jagdale, Vutukuru Kalyan Kumar Reddy, Mohith Prakash Kondapalli, Saimounika Adapa, Diksha Sabharwal

**Affiliations:** 1 General Medicine, Dr. D. Y. Patil College, Hospital and Research Centre, Dr. D. Y. Patil Vidyapeeth, Pune, IND

**Keywords:** abdominal cect, biopsy, extrapulmonary manifestation, hepatic tuberculosis (hepatic tb), tuberculosis (tb)

## Abstract

Millions of people around the world suffer from tuberculosis (TB), a long-term contagious illness. TB can invade almost all human body systems, with the respiratory system being the most impacted. Hepatic TB is a form of TB infection that affects the liver. It is considered an extrapulmonary manifestation of TB, which is a rare manifestation. Early identification of hepatic TB allows for prompt treatment, while leaving it untreated can result in fatality. Our patient is a 46-year-old female who presented with fever, weight loss, loss of appetite, and abdominal pain. An abdominal contrast-enhanced computed tomography (abdominal CECT) scan shows a well-defined, peripherally enhancing hypodense lesion in the liver, which helps with diagnosis. The biopsy demonstrates granulomatous inflammation accompanied by caseating necrosis. The objective of our study is to provide a detailed description of this unique condition through a case presentation.

## Introduction

Tuberculosis (TB) is a widespread health issue in India, affecting approximately 40% of the population, with 2.5 million cases currently active. While the lungs are the primary target, the disease can impact any organ. Around 15-20% of individuals without immune deficiencies exhibit extrapulmonary involvement, which increases to over 50% in those with human immunodeficiency virus (HIV) infection. The country faces a tremendous problem in terms of public health due to the high prevalence of TB. Extrapulmonary TB can exhibit symptoms that can mimic diverse inflammatory and neoplastic disorders, aside from infectious conditions. A high level of clinical suspicion is crucial, especially in countries where TB is endemic [1].

TB exists worldwide, with a 95% prevalence in impoverished nations. TB cases in affluent countries have increased due to immigration from high-TB countries, drug addiction, and HIV infection. Only 3.5% of extrapulmonary TB patients have abdominal TB, and hepatic TB is unusual [2].

Hepatic TB presents challenges in diagnosis due to the absence of typical clinical symptoms and imaging findings, often leading to misdiagnosis and delayed treatment. The literature predominantly consists of case reports, reflecting a limited understanding and experience in diagnosing and treating hepatic TB. The current study contributes by describing a case involving hepatic TB [3].

## Case presentation

A female patient, 46 years old, presented to our medical center with complaints of intermittent fever for two months and mild abdominal pain located in the epigastrium and the right upper quadrant for one month. The patient also complained of loss of appetite and noted weight loss of around 10-12 kg in the last two months. She was not suffering from any comorbidities.

A general examination revealed a poor build and malnutrition, with a body mass index of 16. She was afebrile with a pulse rate of 98 beats per minute, a blood pressure of 106/60 mmHg, respiration of 24 cycles/minute, and an oxygen saturation of 95% in room air. Pallor was present, along with cervical lymphadenopathy (measuring 2x1 cm on the right side and 1x1 cm on the left side) and inguinal lymphadenopathy (measuring 4x3 cm on the right side and 2x1 cm on the left side).

During the systemic evaluation, we noted moderate tenderness over the right upper quadrant associated with hepatomegaly on the per-abdomen examination. Routine blood tests came back normal except for mild normocytic anemia, leucocytosis, elevated erythrocyte sedimentation rate, and elevated alkaline phosphatase levels (Table 1). The chest X-ray was normal.

**Table 1:** Routine blood investigations. SGOT: serum glutamic-oxaloacetic transaminase; SGPT: serum glutamic pyruvic transaminase; ALP: alkaline phosphatase; T3: triiodothyronine; T4: thyroxine; TSH: thyroid-stimulating hormone; ESR: erythrocyte sedimentation rate; RBS: random blood sugar; HIV: human immunodeficiency virus

Parameters	Result	Reference range
Haemoglobin	11 gm/dL	12-16 gm/dL
Total leucocyte count	11,600/μL	4,000-10,000/µL
Platelet count	2,32,000/μL	1,50,000-4,10,000/µL
Serum urea	38 mg/dL	17-49 mg/dL
Serum creatinine	0.62 mg/dL	0.6-1.35 mg/dL
Serum total bilirubin	1.1 mg/dL	0.2-1.2 mg/dL
Conjugated bilirubin	0.3 mg/dL	0.2-0.3 mg/dL
Unconjugated bilirubin	0.8 mg/dL	0.1-1 mg/dL
SGOT	32 IU/L	8-48 IU/L
SGPT	40 IU/L	7-55 IU/L
ALP	156 IU/L	35-104 IU/L
Serum sodium	138 mmol/Lt	136-145 mmol/Lt
Serum potassium	3.6 mmol/Lt	3.5-5.1 mmol/Lt
T3	0.7 ng/mL	0.64-1.52 ng/mL
T4	5.87 ng/mL	4.87-11.72 ng/mL
TSH	3.93 ng/mL	0.35-4.94 ng/mL
ESR	75 mm/hr	Up to 20 mm/hr
RBS	110 mg/dL	Up to 140 mg/dL
HIV antibody	Negative	
Hepatitis B antibody	Negative	
Hepatitis C antibody	Negative	

**Figure 1:**
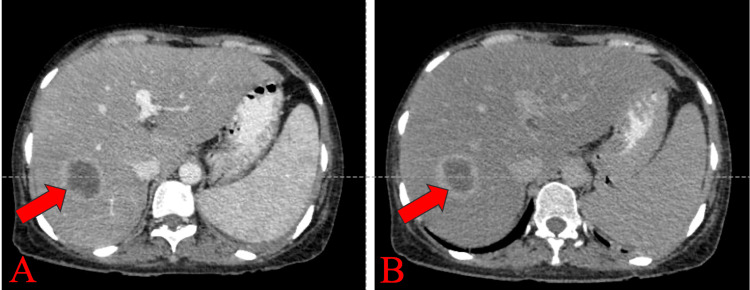
CECT-AP axial section: (A) venous phase showing a well-defined hypodense lesion with peripheral enhancement in segments VII and IV of the liver (shown by the red arrow) likely to be a hepatic abscess and (B) delayed phase showing the same lesion with peripheral enhancement shown by the red arrow. CECT-AP: contrast-enhanced computed tomography of the abdomen and pelvis

**Figure 2:**
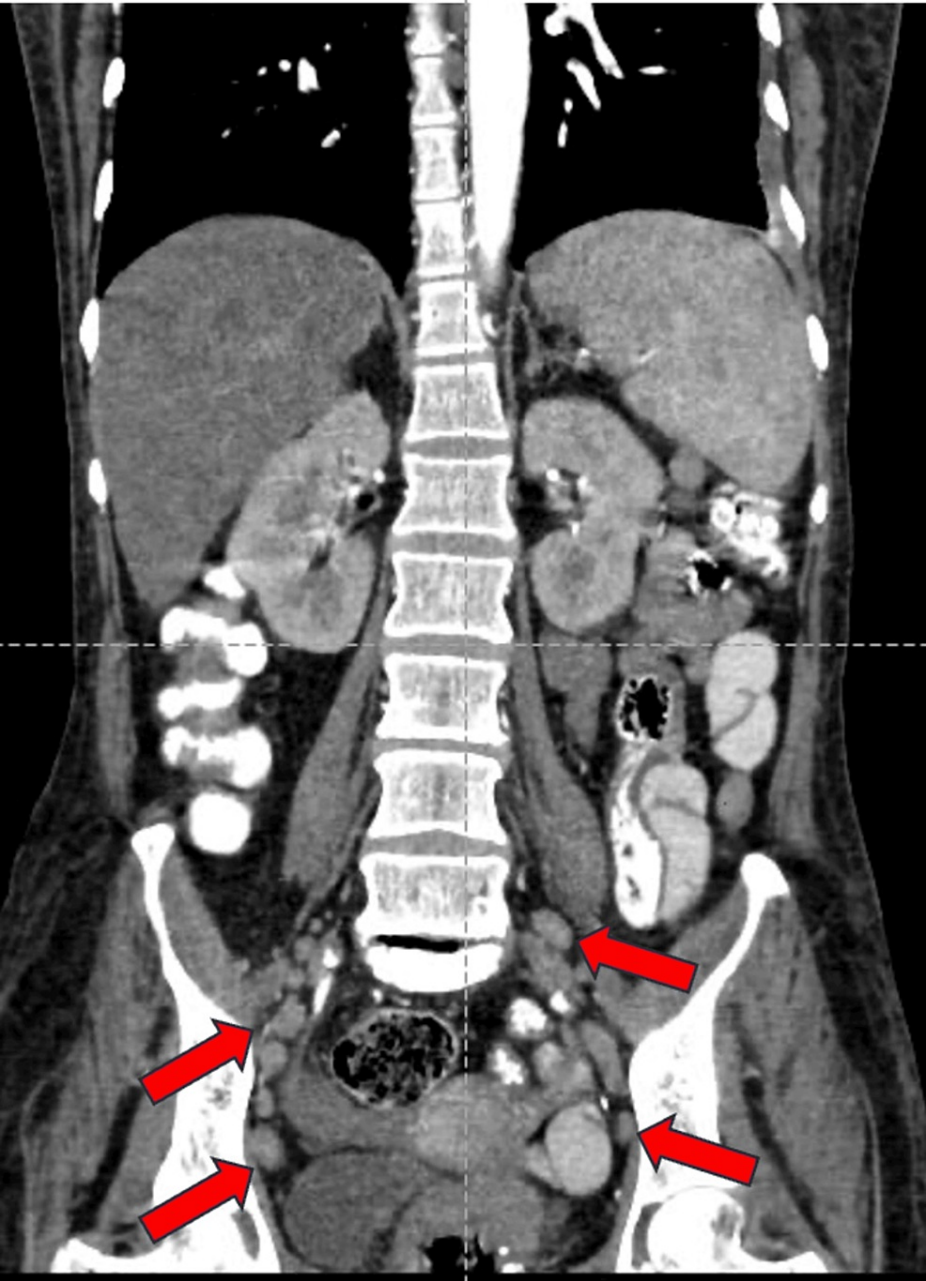
CECT-AP reformatted coronal section showing enhancing lymph nodes along bilateral iliac vessels shown by the red arrows. CECT-AP: contrast-enhanced computed tomography of the abdomen and pelvis

**Figure 3:**
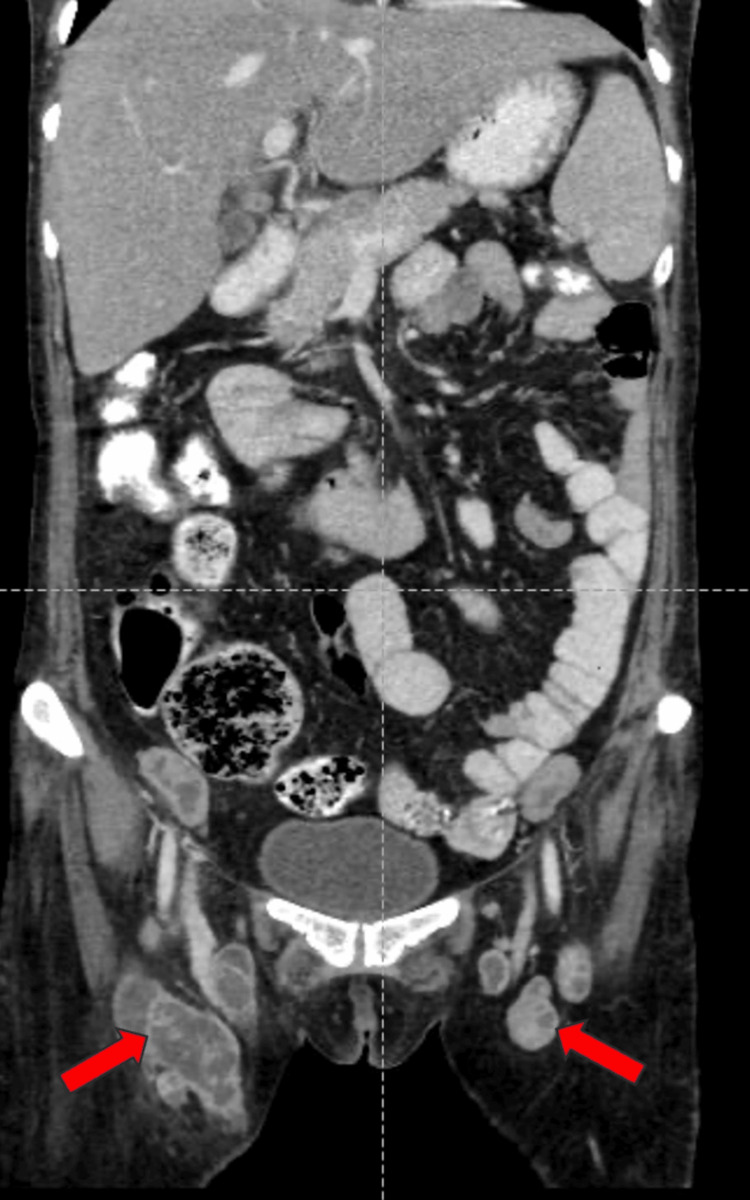
CECT-AP reformatted coronal section showing non-enhancing necrotic lymph nodes in the bilateral inguinal region shown by the red arrows. CECT-AP: contrast-enhanced computed tomography of the abdomen and pelvis

**Figure 4:**
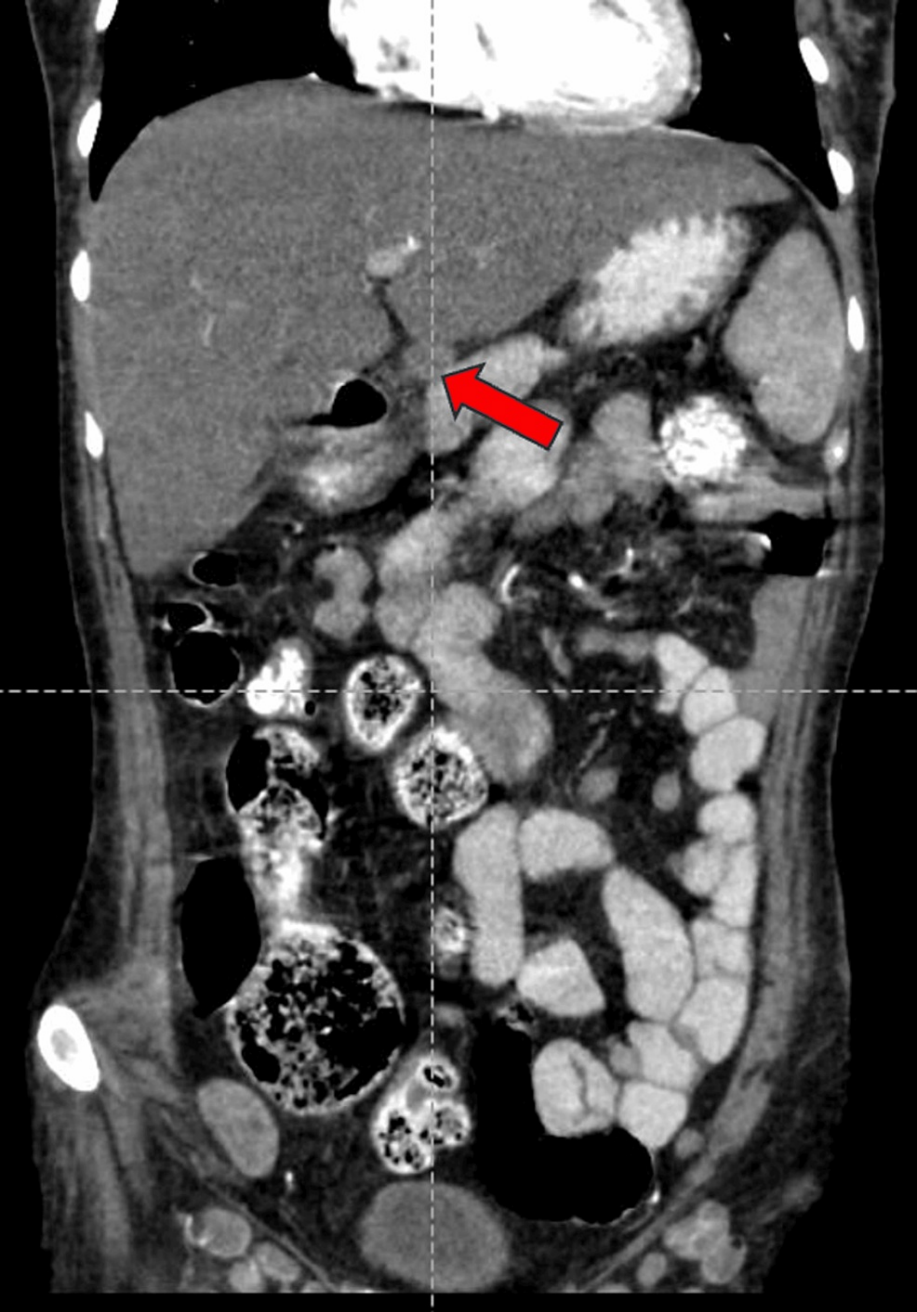
CECT-AP reformatted coronal section showing non-enhancing necrotic at the porta hepatis shown by the red arrow. CECT-AP: contrast-enhanced computed tomography of the abdomen and pelvis

**Figure 5:**
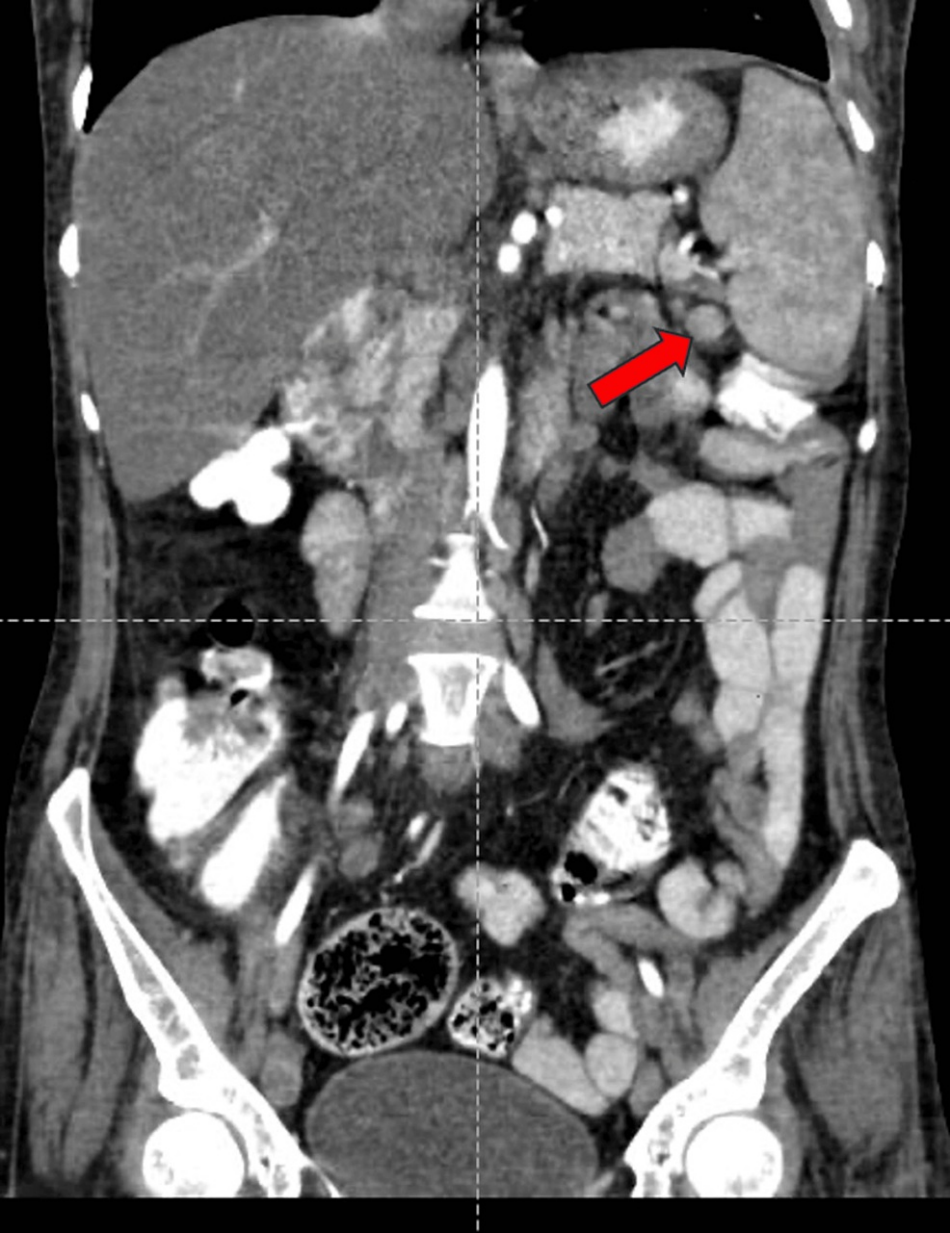
CECT-AP reformatted coronal section showing an enhancing lymph node at the splenic hilum shown by the red arrow. CECT-AP: contrast-enhanced computed tomography of the abdomen and pelvis

**Figure 6:**
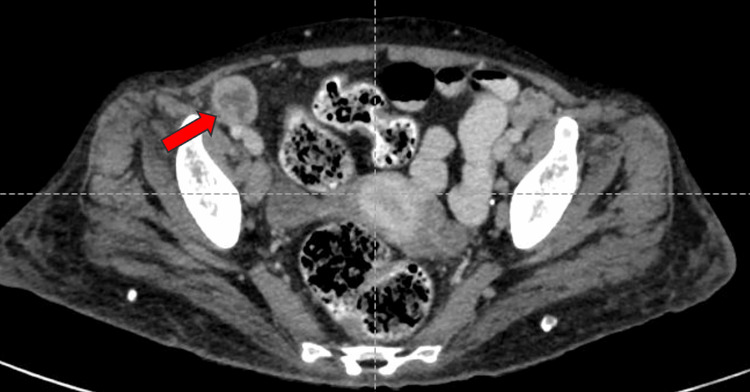
CECT-AP axial section showing an enlarged, non-enhancing necrotic lymph node along the right iliac vessels (right obturator node) shown by the red arrow. CECT-AP: contrast-enhanced computed tomography of the abdomen and pelvis

A well-defined, peripherally enhancing hypodense lesion measuring 34x36x32 mm (CCxAPxTR) was noted in segments VII and VI of the right lobe of the liver on contrast-enhanced computed tomography of the abdomen and pelvis (CECT-AP). It coalesced with a similar sub-centimetric-sized lesion just inferior to it, most likely suggestive of a liquefied hepatic abscess (Figures 1A-1B). We noted multiple enlarged centrally necrotic discrete and few coalescing lymph nodes at the splenic hilum, porta hepatis, upper abdomen, and retroperitoneum, along bilateral iliac vessels, bilateral obturator region, and bilateral inguinal region (Figures 2, 3, 4, 5, 6). Findings suggested an infectious disease. TB needs to be ruled out. We induced sputum by nebulizing it with a 3% hypertonic saline solution and sent it for sputum studies (Table 2). The sputum samples did not show any acid-fast bacilli (AFB).

**Table 2:** Sputum studies. CBNAAT: cartridge-based nucleic acid amplification test; C/S: culture sensitivity; MTB: *Mycobacterium tuberculosis*

Test	Report
Sputum routine microscopy	No acid-fast bacilli
Sputum C/S	No growth
Sputum CBNAAT	MTB not detected

**Figure 7:**
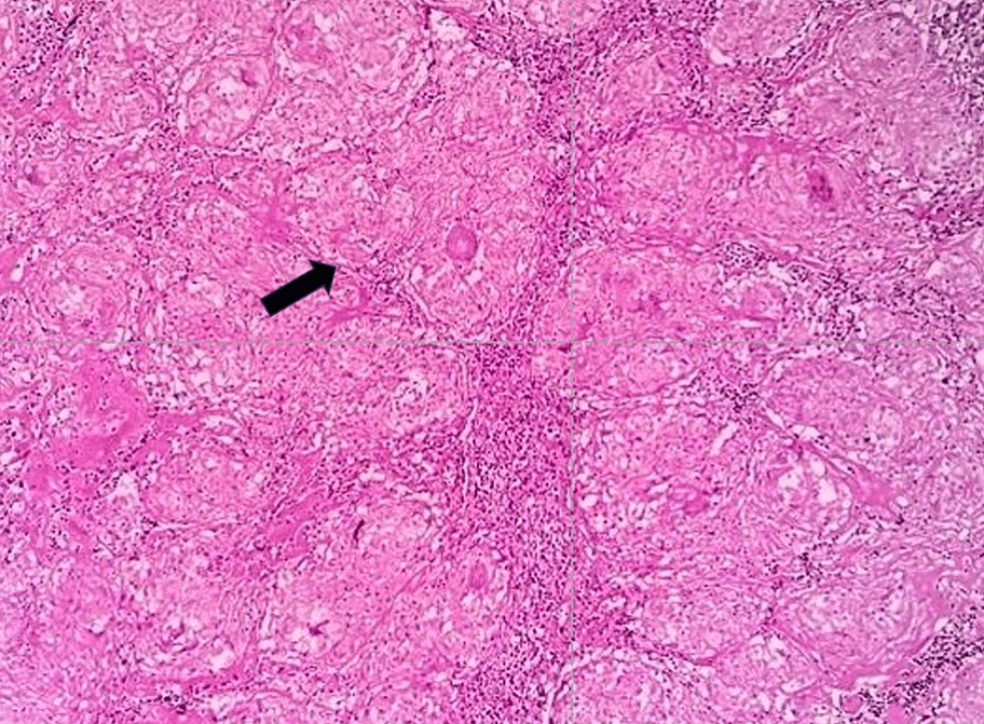
Inguinal lymph node section showing multiple epithelioid cell granulomata with surrounding caseating necrosis under 20x magnification H&E stain. H&E: haematoxylin and eosin

**Figure 8:**
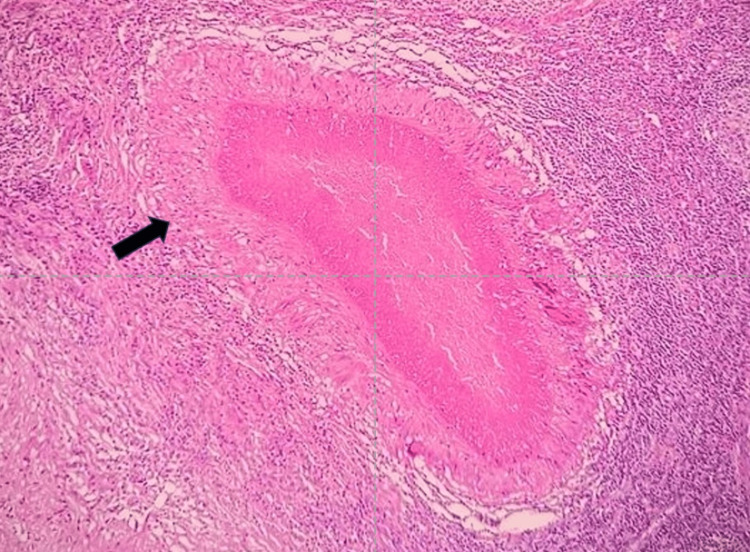
Inguinal lymph node section showing the central area of caseous necrosis with surrounding lymphohistiocytes and Langhans-type giant cells and the peripheral rim of lymphocytes under 40x magnification H&E stain. H&E: haematoxylin and eosin

After the radiological investigation mentioned above, we performed ultrasonography (USG)-guided fine-needle aspiration cytology (FNAC) to extract pus from a hepatic abscess. Additionally, the patient underwent surgical removal of the right inguinal lymph node for biopsy with the use of a local anaesthetic. We sent both the pus specimen from the hepatic abscess and the lymph node samples from the inguinal area for culture sensitivity (C/S) and cartridge-based nucleic acid amplification test (CBNAAT). The CBNAAT of both specimens showed the presence of *Mycobacterium tuberculosis* (MTB), which was sensitive to rifampicin. However, the C/S tests for both specimens did not detect any microorganisms. We sent an inguinal lymph node biopsy specimen for histopathological examination, which revealed multiple epithelioid cell granulomata with surrounding caseating necrosis under 20x magnification haematoxylin and eosin (H&E) stain (Figure 7), the central area of caseous necrosis with surrounding lymphohistiocytes and Langhans-type giant cells, and the peripheral rim of lymphocytes under 40x magnification H&E stain (Figure 8).

We sent an *Entamoeba histolytica* immunoglobulin G (IgG) enzyme-linked immunosorbent assay (ELISA) and a hepatic abscess pus aspiration for C/S to rule out other common causes of liver abscesses, such as an amoebic liver abscess or a pyogenic liver abscess. Both tests came back negative (Table 3).

**Table 3:** Additional investigations. CBNAAT: cartridge-based nucleic acid amplification test; C/S: culture sensitivity; MTB: *Mycobacterium tuberculosis*; IgG: immunoglobulin G; ELISA: enzyme-linked immunosorbent assay

Test	Report
Hepatic abscess pus aspiration C/S	No growth
Hepatic abscess pus aspiration CBNAAT	MTB detected
Inguinal lymph node C/S	No growth
Inguinal lymph node CBNAAT	MTB detected
*Entamoeba histolytica* IgG ELISA	Negative

Comprehensive investigations established a diagnosis of hepatic TB manifested as a hepatic abscess. Two months of anti-tubercular therapy consisted of a combined regimen of rifampicin (10 mg/kg daily), isoniazid (5 mg/kg daily), ethambutol (15 mg/kg daily), and pyrazinamide (25 mg/kg daily); the patient then had four months of anti-tubercular therapy with rifampicin and isoniazid alone. The therapy was completed without any significant adverse effects, and all complaints and laboratory data returned to their baseline levels. The follow-up has been uneventful.

## Discussion

The infection of MTB, an aerobic bacillus, causes TB and affects both the lungs and systemic organs. The WHO considers it a major worldwide public health issue, affecting roughly a third of the global population and causing 1% of new infections annually [3,4].

Hepatic TB is extremely rare, representing barely 1% of all TB illnesses. The scarcity of this occurrence can be attributed to the diminished oxygen pressure in the liver tissue, which obstructs the proliferation of aerobic microorganisms. Frequently observed symptoms and indicators comprise pain in the abdomen, mild fever, hepatomegaly, and right upper quadrant tenderness. The patient showed each of the aforementioned characteristics. Young adults are the most commonly affected age group, although this condition can affect individuals of almost any age [5,6].

Hepatic TB may manifest either as a primary condition or as a secondary infection resulting from TB in other localized areas. The occurrence of miliary disease affecting the liver arises when TB bacilli infiltrate the tissue through the hepatic artery, typically originating from pulmonary TB, but not in this case scenario. Liver invasion through the portal vein can occur, particularly when the gastrointestinal tract is affected. Hepatic TB typically occurs through the portal vein pathway in the liver. Bacteria can also invade the liver through the lymphatic drainage system or through the rupture of lymph nodes containing bacilli within the portal canal. Irrespective of the point of entrance, the liver reacts to this intrusion by generating granuloma tissue [7,8].

Because the liver has numerous reticuloendothelial cells and a lot of blood flow and is at the far end of the portal circulation, granulomata often form there. Its ample vascular perfusion, positioning near the terminal segment of the portal circulation, and significant presence of reticuloendothelial cells contribute to this. Due to the fact that most granulomata are situated in close proximity to the portal canal and have only a mild impact on liver function, the majority of patients experience minimal or no symptoms [9].

Even when the infection is limited to the ipsilateral lobe, contralateral lobe reactivation can make treating hepatic TB challenging. Prompt detection and treatment are critical to preserving remaining liver function [5,8]. The early detection of extrapulmonary TB is difficult, emphasizing the need for a high level of clinical suspicion. A biopsy of the liver, radiological imaging, and histology study confirm the diagnosis, but postponing treatment can cause liver failure and mortality. In the majority of cases, early detection and management with anti-tuberculosis regimens, including medications like isoniazid, rifampicin, pyrazinamide, and ethambutol, result in a favorable prognosis, as we saw in our case.

## Conclusions

Hepatic TB presents with unspecified clinical manifestations, creating diagnostic challenges. The combination of imaging modalities and computed tomography (CT)/USG-guided FNAC proves valuable in diagnosis. An abdominal CECT is a preferred imaging modality that consistently reveals a well-defined, peripherally enhanced hypodense lesion in the liver, aiding in diagnosis, along with granulomatous inflammation accompanied by caseating necrosis on histopathological examination. Hepatic TB is often overlooked unless there is a high suspicion of TB due to its non-specific symptoms. Early detection of hepatic TB allows for efficient treatment and management, whereas failure to treat it might result in death.
